# What Is the Most Effective Strategy for Acute Postoperative Pain in Total Knee Arthroplasty—Retrospective Observational Study

**DOI:** 10.3390/jcm14228138

**Published:** 2025-11-17

**Authors:** Jin Joo, Man Soo Kim, Jeha Lee, Hyun Jung Koh

**Affiliations:** 1 Department of Anesthesiology and Pain Medicine, Seoul St. Mary’s Hospital, College of Medicine, The Catholic University of Korea, Seoul 06591, Republic of Korea; jiyo1004@catholic.ac.kr (J.J.); jeha98@songeui.ac.kr (J.L.); 2 Department of Orthopedics, Seoul St. Mary’s Hospital, College of Medicine, The Catholic University of Korea, Seoul 06591, Republic of Korea; kms3779@naver.com

**Keywords:** total knee arthroplasty, robot-assisted, remimazolam, patient-controlled analgesia

## Abstract

***Background and Objectives:*** Effective early postoperative pain control is essential for optimal recovery following total knee arthroplasty (TKA). In addition to pharmacological pain management, the choice of anesthetic agents and surgical technique can significantly impact postoperative outcomes. Remimazolam and robotic-assisted TKA (RA-TKA) have recently gained attention due to their potential advantages. This study aims to evaluate the effects of remimazolam and RA-TKA on acute postoperative pain compared with conventional TKA (C-TKA) and standard anesthetic protocols. ***Materials and Methods:*** In this retrospective observational study, 460 patients undergoing elective unilateral TKA were divided in to four groups based on surgical technique and anesthetic agents; RA-TKA with remimazolam (Group RR, n = 115), C-TKA with remimazolam (Group CR, n = 134), RA-TKA with conventional anesthesia (Group RC, n = 79), and C-TKA with conventional anesthesia (Group CC, n = 152). Remimazolam was administered at 6 mg/kg/h for induction and 1 mg/kg/h for maintenance, whereas conventional anesthesia was induced with propofol (1.5 mg/kg) and maintained with sevoflurane (1.5~2.0 vol%). The primary endpoint was acute postoperative pain, assessed via patient-controlled analgesia (PCA) consumption and supplemental analgesic use on the day of surgery. Secondary endpoints included total PCA consumption and additional analgesic use during the first 72 h, recovery room stay, discharge scores, and the incidence of postoperative nausea and vomiting (PONV). ***Results:*** Group RR required significantly fewer additional analgesics on the day of surgery than the other groups. Although group RR and group CR exhibited prolonged recovery room stay and lower discharge scores, these outcomes were not correlated with PCA consumption or supplementary analgesic use. RA-TKA was associated with approximately a 31% reduction in additional analgesic use compared with C-TKA, indicating a major contribution of the surgical technique to early postoperative pain control. Remimazolam alone did not demonstrate an independent effect on acute pain management. ***Conclusions:*** RA-TKA combined with remimazolam significantly reduced the need for additional analgesics on the day of surgery, highlighting a synergistic effect of the anesthetic and surgical approach. These findings support RA-TKA with remimazolam as an effective strategy for managing early postoperative pain following TKA.

## 1. Introduction

Early postoperative pain, typically occurring within the first 24 to 72 h after surgery [1], the most severe pain phase and plays a crucial role in determining postoperative recovery and rehabilitation outcomes. Inadequate pain control during this period can delay ambulation, prolong hospitalization, and increase the risk of chronic pain development. Therefore, effective management of acute postoperative pain is a fundamental component of perioperative care, particularly in procedures associated with substantial pain, such as total knee arthroplasty (TKA). To address this, a variety of multimodal analgesic strategies have been developed and continuously refined [2,3,4].

In recent years, growing attention has been directed toward intraoperative pain control rather than focusing solely on postoperative interventions. Effective pain management depends not only on postoperative nerve blocks and analgesics but also on the choice of intraoperative anesthetic agents and surgical techniques. Among recently developed anesthetics, remimazolam, an ultra-short-acting benzodiazepine, has attracted increasing clinical interest due to its unique pharmacological properties [5,6]. Remimazolam combines the rapid onset and offset characteristics of remifentanil with the pharmacodynamic profile of midazolam, offering hemodynamic stability, rapid metabolism, and the availability of a specific reversal agent [7]. Although its direct analgesic effects remain uncertain, several studies have reported that remimazolam may reduce postoperative pain intensity and opioid consumption in both pediatric and adult populations [8,9,10]. Nevertheless, results remain inconsistent owing to variations in study design and assessment methods.

Currently, robot-assisted surgery has been increasingly adopted across various surgical fields to perform more precise and effective procedures. In orthopedic surgery, the use of robot-assisted TKA (RA-TKA) has been introduced and expanded simultaneously, offering greater precision and consistency compared with conventional techniques [11,12,13] and potentially improving postoperative recovery.

Nonetheless, considerable variability exists in the timing and methods used to assess postoperative pain and the effectiveness of these interventions.

Robotic systems enhance the accuracy of bone resection, and soft tissue balancing, minimize intraoperative neurovascular injury and blood loss, and have been associated with improved functional outcomes and patient satisfaction [13,14,15,16,17]. several studies have suggested that RA-TKA may contribute to reduce early postoperative pain compared with conventional TKA [18,19,20], although findings remain controversial [21].

Given these developments, a better understanding of how intraoperative factors—including anesthetic agents and surgical techniques—affect early postoperative pain following TKA is essential for optimizing perioperative care.

Therefore, the present retrospective observational study aimed to evaluate the effects of remimazolam anesthesia and robot-assisted surgical techniques on acute postoperative pain after TKA, compared with conventional anesthetic agents and standard procedures. The findings are expected to provide evidence-based insights into the optimal combination of anesthetic and surgical strategies to improve postoperative pain control and recovery outcomes in clinical practice.

## 2. Methods

### 2.1. Ethical Considerations

This retrospective, observational study was approved by the Ethics Committee of Seoul St. Mary’s Hospital (KC25RISI0133) on 11 March 2025, adhering to the principles of the Declaration of Helsinki. This study was registered with the Clinical Research Information Service, Republic of Korea (http://cris.nih.go.kr accessed on 13 October 2025), KCT0010316) on 19 March 2025. Given the retrospective design of the study, the requirement for informed consent was waived. Following approval from the information Committee for review by the Data Review Committee (DRC, Approval no. 20250131-057), patient information and research data were obtained through a review of electronic medical records (EMRs)

### 2.2. Study Population

**Figure 1:**
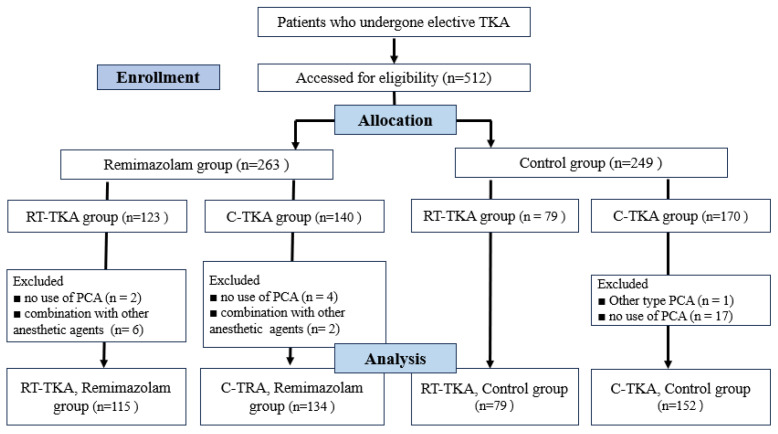
Flow diagram. Abbreviations: PCA, Patient-Controlled Analgesia; RT-TKA, robot-assisted total knee arthroplasty; C-TKA, conventional total knee arthroplasty.

This study included adult patients with an American society of Anesthesiology physical status (ASA-PS) I to III who underwent unilateral TKA between September 2023 and February 2025. Data were collected through electronic medical records. Among these patients, patients who have a history of postoperative nausea and vomiting (PONV), two or more risk factors for PONV, previously experienced adverse effects from patient-controlled analgesia (PCA), or underwent unilateral bur revisional TKA were excluded. A total of 512 patients were initially enrolled and were divided into two groups according to main anesthetic agents: the group receiving remimazolam (n = 263) and the group receiving conventional anesthesia with propofol and sevoflurane (n = 249). The initial enrolled patients were further stratified based on the surgical technique, yielding four groups; Group RR (RA-TKA with remimazolam, n = 123), Group CR (C-TKRA with remimazolam, n = 140), Group RC (RT-TKRA with conventional anesthesia, n = 79) and Group CC (C-TKRA using conventional anesthesia, n = 170). Patients were excluded if they (1) used a different type of PCA or did not use PCA while the devices were routinely prepared, (2) received an alternative PCA that prevented accurate quantification of analgesic use, or (3) were administered supplementary anesthetic agents in addition to the primary anesthetics, such as propofol or inhalational agents in combination with remimazolam. Consequently, the final study population comprised 480 patients; Group RR (n = 115), Group CR (n = 134), Group RC (n = 79) and Group CC (n = 152) (Figure 1).

### 2.3. Study Protocol

This study was conducted as retrospective observational study; therefore, no standardized criteria for the selection of surgical and anesthetic techniques were established. Patients underwent surgery according to their regular schedule. All TKA procedures were performed by the same surgeon using a consistent surgical technique, with assisting medical staff following standardized protocols for all perioperative processes. Anesthesia was administered by orthopedic anesthesia specialists according to a standardized protocol.

The choice of surgical method, however, was determined based on the availability of sterilized robotic instruments and the discretion of the operating surgeon. All surgeries were performed by two experienced orthopedic surgeons with comparable proficiency. The RA-TKA technique was adopted simultaneously by both surgeons at the time of its introduction. Throughout the study period, there were no significant differences between the two surgeons in terms of surgical experience, operative volume, or operation time. Anesthesia was assigned by two anesthesiologists, who independently selected either remimazolam or a standard anesthetic regimen.

All preoperative analgesics were discontinued upon admission on the day before surgery. Although all enrolled patients had been receiving analgesics prescribed by the orthopedic department, none included opioid medications. On the day of surgery, patients were instructed by the surgical team to take celecoxib 200 mg, a selective COX-2 inhibitor, two hours prior to surgery to reduce inflammation and alleviate pain.

#### 2.3.1. Anesthetic Techniques

Except for the main anesthetic agent, all other anesthetic methods were standardized. For induction, after initial infusion of remimazolam at 6 mg/kg/h for 3 min, anesthesia was maintained with a continuous infusion at 1 mg/kg/h. In control group, anesthesia was induced with 1% propofol 1.5 mg/kg and maintained with sevoflurane at 1.5~2 vol%. Rocuronium (0.8 mg/kg) was administered as a neuromuscular blocker, and remifentanil (2 to 3 ng/mL) was administered via target-controlled infusion as an opioid. Fresh gas flow was maintained with O_2_ (1.5 L/min) and air (2.5 L/min) until the end of the surgery. Apart from the administration of 20 mg of rocuronium at the time of tourniquet deflation, no additional anesthetic agents were administered for maintenance. At the end of the surgery, 200 mg of sugammadex was administered. Extubation was performed after confirming the return of spontaneous respiration, a Bispectral Index (BIS) value > 85, and a Train-of-Four (TOF) ratio > 95%. As in the previous report [22], flumazenil 0.3 mg was administered intravenously to patients treated with remimazolam if emergence from anesthesia was delayed, defined a drowsy state with Ramsay sedation score above 3.

Patients were transferred to the Post-Anesthesia Care Unit (PACU) and observed for 40 min, which represents the standard recovery room stay time at our institution. During the PACU stay, nausea and vomiting, pain scores, and Modified Aldrete score at both admission to and discharge from the PACU were assessed. Patients were transferred to the ward when the Modified Aldrete Score was >8, including a respiratory score 2. Postoperative pain was primarily managed using patient-controlled analgesia (PCA), consisting of fentanyl 500 μg, nefopam hydrochloride 100 mg, and ramosetron hydrochloride 0.3 mg, with a basal rate of 1 mL, bolus of 1 mL, and a lockout time of 10 min, maintained for up to 72 h after surgery. PCA use was initiated in the PACU as part of standard protocol and was maintained throughout the early postoperative phase, defined as the period from PACU admission to 72 h after surgery, unless interrupted due to adverse effects. In the PACU, fentanyl 50 µg was administered intravenously (i.v.) as a bolus, and if inadequate analgesia persisted, additional analgesic boluses were administered at 15 min intervals, up to a maximum three doses. If pain persisted despite these doses, PRN analgesics were administered on the ward after PACU discharge according to the ward’s pain management protocol. Additional analgesics were administered whenever the visual analoque scale (VAS) exceeded 7, with the target of maintaining a VAS ≤ 3. On the word, pethidine 50 mg and tramadol 100 mg were administered alternately as needed. For the management of postoperative nausea and vomiting (PONV), ondansetron 4 mg was administered in the PACU, while palonosetron 0.75 mg was given on the ward. To facilitate early postoperative pain control and rehabilitation, PCA was maintained for up to 72 h postoperatively in the patients undergoing TKA and then discontinued. All patients were discharged on postoperative day (POD) 5 without complications.

#### 2.3.2. Surgical Techniques

All operation were performed by two surgeons with comparable surgical proficiency. C- TKAs were performed using a standardized technique. After the introduction of RA-TKA, the procedure was alternated with C-TKA depending on the surgical schedule, as daily equipment sterilization limited the availability of the robotic system. RA-TKA was performed using the Stryker MAKO^®^ Robotic-Arm Assisted Surgery System (Stryker Corp., Mahwah, NJ, USA), which is a semi-automated platform. No additional procedures, such as periarticular infiltration for intraoperative or postoperative pain control, were performed.

### 2.4. Clinical Variables

#### 2.4.1. Primary Outcome

The primary outcomes of this study were the total PCA consumption on the day of surgery (postoperative day 0) and the number of supplemental analgesic administrations.

#### 2.4.2. Secondary Outcomes

The secondary outcomes included all variables assessing early postoperative pain excluding the primary outcomes. Specifically, total PCA consumption, daily PCA use, the frequency of PRN analgesic administration, and the incidence of PONV and use of antiemetic medications were evaluated and compared on postoperative days 1 and 2.

### 2.5. Statistical Analyses

Patients were classified into four groups for comparison. Continuous variables were analyzed using one-way analysis of variance (ANOVA) for parametric data and the Kruskal–Walli’s test for nonparametric data, while categorical variables were compared using the chi-squared test. For outcomes expressed as counts such as the number of rescue analgesic dose and postoperative nausea and vomiting, statistical models appropriate for count data were additionally applied. When significant overall differences were observed, post hoc pairwise comparisons were conducted with appropriate adjustments. To adjust for potential confounders, including sex, analysis of covariance (ANCOVA) or multiple regression analysis was conducted. Interaction terms were prespecified to assess the combined effects of anesthesia type and surgical method. Data are presented as mean ± standard deviation (SD) for continuous variables and as number (percentage) for categorical variables. A *p*-value of < 0.05 was considered statistically significant. All statistical analyses were conducted using SPSS version 22 (IBM Corp., Armonk, NY, USA), and figures were generated using Microsoft Excel (Microsoft Corp., Redmond, WA, USA).

## 3. Results

### 3.1. Demographic Characteristics

#### 3.1.1. Preoperative Characteristics

There were no significant differences in mean age or body mass index (BMI) among the four groups (*p* = 0.257 and *p* = 0.060, respectively). The proportion of female patients was higher across all groups (female, 410 [85.4%]; male, 70 [14.6%]), and the difference in gender distribution was statistically significant (*p* = 0.013). Regarding the American Society of Anesthesiologists Physical Status (ASA-PS) classification, most patients in all groups were classified as ASA-PS II, with no significant intergroup difference observed (*p* = 0.163) (Table 1).

#### 3.1.2. Intraoperative Variables

Operation time differed significantly among the four groups (*p* < 0.001). Post hoc comparisons revealed that operation time was significantly longer in group RR than in group CR (*p* = 0.003) and in group RC than in group CR (*p* = 0.002), whereas no significant difference was observed between group RR and group RC (*p* > 0.05).

Anesthesia time also showed a significant difference among groups (*p* < 0.001). Post hoc analysis indicated that group CR had a significantly shorter anesthesia duration compared with groups RR (*p* < 0.001) and RC (*p* = 0.002).

Although remifentanil consumption tended to be highest in group RR and lowest in group CC, there was no statistically significant difference in the total remifentanil dose among the four groups (*p* = 0.426).

#### 3.1.3. Postoperative Variables

The post-anesthesia care unit (PACU) stay was significantly longer in patients who received remimazolam (*p* < 0.001). These findings indicate that the choice of anesthetic agent has a considerable impact on recovery time, which is an important determinant of PACU duration. Even when the same surgical technique was used, PACU stay remained significantly longer in the remimazolam groups (group CR vs. CC, *p* = 0.022; group RR vs. RC, *p* < 0.001). Furthermore, Modified Aldrete scores assessed immediately before discharge from PACU showed significantly lower values in the remimazolam groups (group CR and group RR) compared with the corresponding controls (*p* < 0.001).

### 3.2. Comparison of PCA Dosage Among Groups

Total PCA consumption, evaluated as an indicator of early postoperative pain control, showed no significant differences among the four groups during the entire PCA administration period. Although PCA usage fluctuated over time within each group, intergroup comparisons at each postoperative day (POD 0 and POD 1) revealed no statistically significant differences (Table 2).

### 3.3. Comparison of Supplemental Analgesic (PRN) Use Among Groups

**Figure 2:**
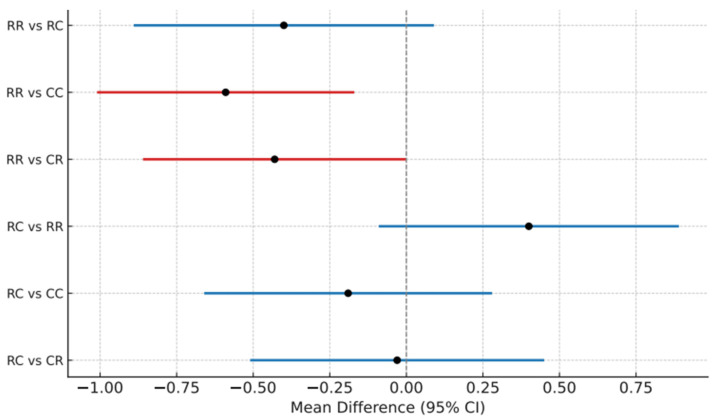
Pairwise comparison of supplemental analgesic use within 24 h among groups.

Comparison of PRN analgesic use at each early postoperative time point revealed significant differences among the groups within the first 24 h after operation (*p* = 0.003). During this period, group RR required significantly fewer PRN analgesic administrations compared with groups CC (*p* = 0.001, 95% CI [−1.01, 0.17]) and CR (*p* = 0.049, 95% CI [−0.85, 0.00]). However, between 24 and 72 h postoperatively, no significant differences in PRN use were observed among the four groups (*p* = 0.413). Consistently, on the day of surgery, group RR demonstrated significantly lower supplemental analgesic requirements than groups CC and CR, whereas no significant intergroup differences were noted thereafter (Table 3 and Figure 2).

In particular, a multivariable regression analysis was conducted to identify factors associated with additional analgesic use on the day of surgery. Neither anesthesia type (OR = 1.12, 95% CI [0.84, 1.48], *p* = 0.454) nor gender (OR = 1.07, 95% CI [0.78, 1.46], *p* = 0.693) was significantly associated with supplemental analgesic requirements. In contrast, the surgical method was the only variable showing a significant association, with robot-assisted surgery linked to an approximately 31% reduction in additional analgesic use within the first 24 h postoperatively (OR = 0.69, 95% CI [0.49, 0.96], *p* = 0.029), suggesting a potential advantage in postoperative pain control. Overall, robot-assisted surgery independently contributed to reduced analgesic consumption, whereas the type of anesthetic agent showed no significant association (OR = 1.24, 95% CI [0.77, 2.01], *p* = 0.371).

### 3.4. Comparison of PONV Occurrence and Antiemetic Use During the Acute Postoperative Period

Following PACU discharge, no significant intergroup differences were observed in PONV incidence or in antiemetic administration at any time point during the acute postoperative period (Table 4).

## 4. Discussion

Considering that effective control of severe early postoperative pain is crucial for functional recovery after TKA, this study identified the effects of remimazolam anesthesia and robot-assisted total knee arthroplasty (RA-TKA) on the management of acute postoperative pain. Our findings demonstrated that robot-assisted surgery was associated with a reduction in additional analgesic requirements during the acute postoperative period, and this effect appeared to be further enhanced when remimazolam was used as the primary anesthetic agent. Although remimazolam alone did not exert a significant independent effect on acute postoperative pain, its interaction with the surgical technique suggested a potential synergistic benefit in optimizing early pain control.

Significant differences were also observed in both anesthesia duration and operative time among the four groups. Notably, patients who underwent conventional TKA under remimazolam anesthesia exhibited shorter surgical and anesthetic times, which may reflect improved procedural efficiency in conventional techniques compared with robot-assisted approaches. However, no significant difference in intraoperative remifentanil consumption was observed across the different TKA approaches, and the opioid dose did not appear to meaningful influence postoperative pain intensity or recovery profiles. These findings suggest that the contribution of intraoperative remifentanil administration to early postoperative outcomes may be limited.

Remimazolam, a short-acting γ-aminobutyric acid type A (GABA*_A_*) receptor agonist, is increasingly favored for its hemodynamic stability and minimal cardiovascular depression. In elderly patients, it has been shown to cause a lower incidence of intraoperative hypotension compared with propofol [23]. Furthermore, during postoperative recovery, remimazolam is associated with a reduced risk of emergence delirium compared with inhalational agents such as sevoflurane [24,25].

Remifentanil, although characterized by rapid plasma metabolism and an ultra-short half-life, has shown inconsistent evidence regarding its analgesic efficacy in the postoperative period. The literature on RA-TKA similarly remains heterogeneous: while several studies report no significant improvement in postoperative pain control [26], and lack of pain alleviation [21], others suggest potential benefits, including reduced pain intensity and earlier functional recovery [18,19,27]. While previous studies have suggested that excessive intraoperative administration of remifentanil may predispose patients to postoperative hyperalgesia [28,29], no significant difference in remifentanil consumption was observed among the different surgical or anesthetic techniques in this study. Therefore, remifentanil use was unlikely to have contributed to the higher postoperative analgesic requirements after surgery.

In the present study, the combination of robot-assisted total knee arthroplasty (RA-TKA) and remimazolam anesthesia was associated with a reduced requirement for additional analgesics during the early postoperative period. This reduction in supplemental analgesic use was not solely attributable to the anesthetic agent itself; rather, the RA-TKA technique appeared to be the primary determinant of improved postoperative pain control. These findings are consistent with previous studies reporting that RA-TKA is associated with reduced short-term postoperative pain and lower opioid requirements [17,18,19]. To date, however, there has been no explicit evidence identifying the combination of RA-TKA and remimazolam anesthesia as a principal determinant of improved postoperative pain control. Therefore, the present results provide novel insight into the potential synergistic interaction between surgical and anesthetic approaches in optimizing postoperative analgesia.

Remimazolam is recognized for its favorable safety profile, particularly in elderly patients, offering a lower incidence of intraoperative hypotension and reduced pharmacodynamic variability owing to its short duration of action [30]. Nevertheless, caution is warranted when remimazolam is co-administered with opioids such as remifentanil, as this combination has been associated with airway-related adverse events [31]. In the present cohort, patients receiving remimazolam demonstrated prolonged PACU stays and lower Modified Aldrete scores at discharge from PACU compared with controls; however, these findings were not attributable to heightened pain levels and did not appear to compromise recovery quality. Moreover, neither the remimazolam dose nor the use of flumazenil was affected. Although remimazolam generally facilitates rapid emergence and minimal residual sedation following flumazenil reversal [32,33], the present study found no significant difference in the amount of flumazenil administered among the different surgical approaches. Therefore, it remains unclear whether the observed prolongation of discharge time was related to the timing or use of flumazenil. Previous studies suggest that remimazolam has limited evidence as a direct analgesic that independently reduces postoperative pain. However, it has been reported to exert an indirect analgesic effect by attenuating pain-related stress responses through sedation at appropriate doses [10,34,35]. In the present study, we could not confirm an independent role of remimazolam in pain reduction, indicating that its potential contribution to postoperative analgesia, particularly after major surgeries such as TKA, warrants further investigation. The acute postoperative period generally encompasses the first 24 to 72 h after surgery and may extend up to 7 days postoperatively [36]. Acute postoperative pain refers to pain occurring this period, with its intensity typically peaking within the initial 24 to 72 h. Inadequate control of pain during this early phase can delay mobilization and functional recovery and increase the risk of complications such as atelectasis and deep vein thrombosis. Furthermore, insufficiently managed acute pain may contribute to the development of chronic postoperative pain through mechanisms of central sensitization. These considerations highlight the critical importance of effective pain management in the early postoperative period, for which various multimodal analgesic strategies have been developed and continuously refined [37].

In this study, although total PCA consumption did not differ among groups, the number of additional analgesic administrations on the day of surgery—when postoperative pain typically peaks—was significantly lower in the robot-assisted TKA group receiving remimazolam compared with all other groups. Considering the pharmacokinetic and pharmacodynamic characteristics of remimazolam and its favorable safety profile in elderly patients [23,38,39], its use may provide distinct advantages in RA-TKA, which predominantly involves an older patient population, particularly when it is associated with reduced supplemental opioid requirements. Although remimazolam alone did not demonstrate an independent analgesic effect, its combination with robot-assisted surgery may offer an effective approach to optimize early postoperative pain management.

Furthermore, although the majority of participants in this study were female, sex did not appear to influence the primary outcomes. This finding is consistent with previous reports [40,41,42] indicating that gender had no significant effect on postoperative pain outcomes or analgesic requirements, thereby supporting the reliability of the present results.

In conclusion, this study demonstrates that the interaction between surgical technique and anesthetic agent significantly influences postoperative pain outcomes in TKA. Among the evaluated factors, the adoption of robot-assisted surgery played a more decisive role than the choice of anesthetic in reducing analgesic requirements and improving early postoperative pain control. Given the hemodynamic stability and favorable safety profile of remimazolam, particularly in elderly patients, its combination with robot-assisted TKA may serve as an effective approach to optimize postoperative recovery.

### Limitations

This study has several limitations. Firstly, although female patients predominated in this (cohort) study, sex did not significantly affect the study outcomes. Nevertheless, future prospective, randomized controlled trials (RCTs) are warranted to elucidate the precise impact of sex on postoperative analgesic responses. Second, because RA-TKA and remimazolam anesthesia were introduced at different time points compared with C-TKA and conventional anesthesia, this retrospective design inevitably led to heterogeneity in surgical and anesthetic techniques as well as unequal group sizes. Prospective RCTs are needed to minimize potential bias arising from these differences and to validate the reliability of our findings. Third, this study compared remimazolam with conventional anesthesia involving both propofol and inhalational agents, rather than exclusively among intravenous anesthetics, which limited direct agent-to-agent comparisons. Future investigations should evaluate the analgesic efficacy of remimazolam under standardized anesthetic conditions. Finally, the observation period in this study was limited to 72 h postoperatively to assess acute pain. Evaluating pain scores and analgesic consumption in the post-anesthesia care unit (PACU) immediately after surgery would provide a more comprehensive understanding of early postoperative pain. Additionally, further research should explore the potential associations between anesthetic techniques, surgical approaches, and the development of subacute or chronic postoperative pain.

## 5. Conclusions

This study identified anesthetic and surgical strategies that effectively managed early acute postoperative pain following TKA. While the anesthetic method alone did not significantly reduce pain, the surgical technique demonstrated a distinct analgesic advantage. Notably, the combination of RA-TKA with remimazolam produced a synergistic effect, as evidenced by reduced postoperative analgesic consumption compared with the other groups. This combined approach contributed to decreased acute postoperative pain and facilitated earlier mobilization, suggesting improved recovery quality after TKA. However, given the limited sample size and retrospective design, large-scale prospective randomized studies are warranted to validate these findings and further clarify the interaction between anesthetic agents and surgical techniques in optimizing postoperative outcomes.

## Figures and Tables

**Table 1:** Comparison of demographic characteristics.

Group	Group RR	Group CR	Group RC	Group CC	*p*-Value
*n*	115	134	79	152	
Preoperative variables					
Age (years)	71.62 ± 5.98	72.34 ± 8.26	71.65 ± 6.31	70.72 ± 6.3	0.257
Gender					0.013 *
Male	24	9	13	24	
Female	91	125	66	128	
ASA PS					0.163
I	1	7	5	10	
II	105	120	70	126	
III	9	7	4	16	
BMI (kg/m^2^)	25.9 ± 3.43	25.27 ± 2.8	26.48 ± 3.54	26.07 ± 3.65	0.06
Intraoperative variables					
Operation duration (min)	100.08 ± 33.89	77.75 ± 17.76	101.53 ± 35.58	91.44 ± 35.42	<0.001 *
Anesthesia duration (min)	137.45 ± 37.86	112.08 ± 21.83	131.38 ± 36.78	120.34 ± 40.02	<0.001 *
Remifentanil total dosage (mg)	0.58 ± 0.24	0.49 ± 0.22	0.43 ± 0.20	0.41 ± 0.18	0.426
Postoperative variables					
Length of stay in PACU (min)	59.53 ± 22.74	59.39 ± 19.89	48.47 ± 9.01	52.16 ± 17.22	<0.001 *
PONV	0	0	0	2	0.289
Anti-emetics	0	0	0	1	1
Modified Aldrete score ^1^	7.62 ± 0.78	7.71 ± 0.56	7.8 ± 0.56	7.70 ± 0.54	0.174
Modified Aldrete score ^2^	9.51 ± 0.52	9.53 ± 0.53	9.76 ± 0.43	9.79 ± 0.44	<0.001 *

Abbreviations: ASA-PS, American Society of Anesthesiologists Physical Status; BMI, Body Mass Index; PACU, Post-Anesthesia Care Unit; PCA, Patient-Controlled Analgesia; PONV, Postoperative Nausea and Vomiting. ^1^: PACU admission, ^2^: PACU discharge. Values are expressed as mean (standard deviation) and number (percentage). RR (RA-TKA + remimazolam), CR (C-TKA + remimazolam), RC (RA-TKA + conventional anesthesia), CC (C-TKA + conventional anesthesia). * *p* < 0.05.

**Table 2:** Comparison of PCA consumption among groups.

Postoperative Variables.	Group RR	Group CR	Group RC	Group CC	
Total PCA	57.85 ± 24.90	53.76 ± 26.88	51.75 ± 24.97	55.86 ± 26.41	0.427
PCA dosage on POD 0	10.40 ± 7.31	10.71 ± 7.35	9.92 ± 6.9	10.06 ± 7.08	0.839
PCA dosage on POD 1	39.98 ± 17.91	39.41 ± 16.89	39.39 ± 20.19	38.93 ± 18.09	0.974

Abbreviations: PONV (postoperative nausea and vomiting), PCA (Patient Controlled Analgesia), POD (postoperative day), RR (RA-TKA + remimazolam), CR (C-TKA + remimazolam), RC (RA-TKA + conventional anesthesia), CC (C-TKA + conventional anesthesia).

**Table 3:** Comparison of supplemental analgesic use among groups.

Postoperative Variables	Group RR	Group CR	Group RC	Group CC	
Number of PRN analgesic use on POD 0	1.02 ± 1.04	1.45 ± 1.42	1.42 ± 1.32	1.61 ± 1.28	0.003
Number of PRN analgesic use on POD1–2	1.32 ± 1.50	1.50 ± 1.41	1.71 ± 1.90	1.41 ± 1.74	0.413

Abbreviations: prn (pro re nata, as needed), PCA (Patient-Controlled Analgesia), POD (postoperative day), RR (RA-TKA + remimazolam), CR (C-TKA + remimazolam), RC (RA-TKA + conventional anesthesia), CC (C-TKA + conventional anesthesia). Values are expressed as mean (standard deviation) and number (percentage).

**Table 4:** Comparison of the occurrence of PONV among the groups.

Postoperative Variables	Group RR	Group CR	Group RC	Group CC	
PONV occurrence (no.)	0	0	0	2	0.228
Anti-emetics within 24 h (no.)	0	0	0	1	0.539
Anti-emetics between 24 and 72 h (no.)	72	86	48	115	0.989

Abbreviations: PONV, postoperative nausea and vomiting; PCA, Patient Controlled Analgesia; no, number. RR (RA-TKA + remimazolam), CR (C-TKA + remimazolam), RC (RA-TKA + conventional anesthesia), CC (C-TKA + conventional anesthesia).

## Data Availability

The data supporting the study findings are considered within the article.

## Abbreviation

TKA	total knee arthroplasty
RA-TKA	robot-assisted total knee arthroplasty
C-TKA	conventional TKA
PCA	patient-controlled analgesia
PONV	postoperative nausea and vomiting
